# The Dose-Response Effects of Consuming High Fructose Corn Syrup-Sweetened Beverages on Hepatic Lipid Content and Insulin Sensitivity in Young Adults

**DOI:** 10.3390/nu14081648

**Published:** 2022-04-15

**Authors:** Desiree M. Sigala, Bettina Hieronimus, Valentina Medici, Vivien Lee, Marinelle V. Nunez, Andrew A. Bremer, Chad L. Cox, Candice A. Price, Yanet Benyam, Yasser Abdelhafez, John P. McGahan, Nancy L. Keim, Michael I. Goran, Giovanni Pacini, Andrea Tura, Claude B. Sirlin, Abhijit J. Chaudhari, Peter J. Havel, Kimber L. Stanhope

**Affiliations:** 1 Department of Molecular Biosciences, School of Veterinary Medicine, University of California—Davis, Sacramento, CA 95616, USA; dmsigala@ucdavis.edu (D.M.S.); bettina.hieronimus@mri.bund.de (B.H.); vilee@ucdavis.edu (V.L.); mvnunez@ucdavis.edu (M.V.N.); caaprice@ucdavis.edu (C.A.P.); ybenyam@ucdavis.edu (Y.B.); pjhavel@ucdavis.edu (P.J.H.); 2 Department of Nutrition, University of California—Davis, Sacramento, CA 95616, USA; 3 Institute for Physiology and Biochemistry of Nutrition, Max Rubner-Institut, 76131 Karlsruhe, Germany; 4 Division of Gastroenterology and Hepatology, Department of Internal Medicine, University of California—Davis, Sacramento, CA 95817, USA; vmedici@ucdavis.edu; 5 Department of Pediatrics, School of Medicine, University of California—Davis, Sacramento, CA 95817, USA; andrew.bremer@nih.gov; 6 Department of Chemistry, California State University, Sacramento, CA 95819, USA; clcox@ucdavis.edu; 7 Department of Family and Consumer Sciences, California State University, Sacramento, CA 95819, USA; 8 Department of Radiology, School of Medicine, University of California—Davis, Sacramento, CA 95817, USA; yabdelhafez@ucdavis.edu (Y.A.); jpmcgahan@ucdavis.edu (J.P.M.); ajchaudhari@ucdavis.edu (A.J.C.); 9 Western Human Nutrition Research Center, United States Department of Agriculture, Davis, CA 95616, USA; nancy.keim@usda.gov; 10 The Saban Research Institute, Children’s Hospital Los Angeles, Los Angeles, CA 90027, USA; goran@usc.edu; 11 Metabolic Unit, Institute of Neuroscience, National Research Council (CNR), 35127 Padova, Italy; giovanni.pacini@cnr.it; 12 Liver Imaging Group, Department of Radiology, University of California—San Diego, La Jolla, CA 92093, USA; andrea.tura@cnr.it (A.T.); csirlin@health.ucsd.edu (C.B.S.)

**Keywords:** sugar-sweetened beverages, high-fructose corn syrup, liver fat, insulin sensitivity, lactate

## Abstract

Increased hepatic lipid content and decreased insulin sensitivity have critical roles in the development of cardiometabolic diseases. Therefore, our objective was to investigate the dose-response effects of consuming high fructose corn syrup (HFCS)-sweetened beverages for two weeks on hepatic lipid content and insulin sensitivity in young (18–40 years) adults (BMI 18–35 kg/m^2^). In a parallel, double-blinded study, participants consumed three beverages/day providing 0% (aspartame: *n* = 23), 10% (*n* = 18), 17.5% (*n* = 16), or 25% (*n* = 28) daily energy requirements from HFCS. Magnetic resonance imaging for hepatic lipid content and oral glucose tolerance tests (OGTT) were conducted during 3.5-day inpatient visits at baseline and again at the end of a 15-day intervention. During the 12 intervening outpatient days participants consumed their usual diets with their assigned beverages. Significant linear dose-response effects were observed for increases of hepatic lipid content (*p* = 0.015) and glucose and insulin AUCs during OGTT (both *p* = 0.0004), and for decreases in the Matsuda (*p* = 0.0087) and Predicted M (*p* = 0.0027) indices of insulin sensitivity. These dose-response effects strengthen the mechanistic evidence implicating consumption of HFCS-sweetened beverages as a contributor to the metabolic dysregulation that increases risk for nonalcoholic fatty liver disease and type 2 diabetes.

## 1. Introduction

Approximately 1 in 10 or over 500 million adults worldwide have diabetes with individuals with type 2 diabetes (T2D) representing over 90% of cases [1,2]. Diabetes is one of the largest global health challenges in the 21st century with the number of cases estimated to rise to 700 million by 2045 [1]. The incidence of nonalcoholic fatty liver disease (NAFLD) among adults is also rising [3] with the global prevalence estimated at 25% [4] and U.S. prevalence at 37% [5]. There is a well-documented link between T2D and NAFLD with both being associated with insulin resistance and an increased risk of metabolic syndrome and cardiovascular disease (CVD) [6,7]. T2D has been described as the strongest predictor of NAFLD and driver of disease progression [8]. Likewise, it has been reported that NAFLD is one of the strongest clinical risk factors for T2D [9]. While insulin resistance is involved in this bidirectional relationship, it is unclear whether hepatic insulin resistance develops first and primes the liver for subsequent fat accumulation or if hepatic insulin resistance is primarily a consequence of increased fat accumulation in the liver [10].

Industrialization has brought pronounced changes in human behavior and lifestyle that have contributed to escalating rates of both T2D and NAFLD [11,12]. These changes include the marked increases in the availability of added sugars and low fiber processed foods [13,14]. Data from the National Health and Nutrition Examination Survey showed that processed foods comprised 65% of energy intake and contributed 91% of the energy intake from added sugars [15,16]. Sugar-sweetened beverages (-SBs), primarily sweetened with high fructose corn syrup (HFCS), are a major contributor of added sugars in the U.S. diet, with youth and adults consuming over 140 kcal per day on average [17,18].

Evidence from observational studies have consistently demonstrated positive associations between increased consumption of sugar-SBs and insulin resistance [19], T2D, [20] and NAFLD [21]. Evidence from clinical dietary intervention studies have shown that increased and sustained (+7 days) exposure to sugar-SBs increases liver lipid content [22,23,24,25] and decreases insulin sensitivity [22,24,26,27]. Clinical dietary intervention studies designed to decrease consumption of sugar-SB [28], HFCS [29], or free/added sugars [30,31,32] have led to reductions in hepatic lipid content.

Both HFCS and sucrose contain fructose and glucose; however, clinical [26] and animal studies [33,34] have demonstrated that it is the unique metabolism of fructose that is the key mediator of the relationship between sugar-SB exposure and metabolic dysregulation. Unlike glucose, the initial phosphorylation of fructose is catalyzed by fructokinase. Fructokinase operates independently of hepatic energy status, resulting in unregulated fructose uptake in both the fasted and fed state. Therefore, as much as 85% of the fructose in a sugar-SB will be taken up during its first pass through the liver [35,36]. The resulting fructose overload in the liver increases uric acid and lactate production. Fructose and/or its metabolites induce expression of sterol regulatory element binding protein 1c (SREBP-1c) and carbohydrate response element binding protein (ChREBP) and lead to increased de novo lipogenesis (DNL) [37,38] and apolipoprotein CIII (apoCIII) production [38,39], and decreased fat oxidation [10,40]. Studies in animal models and in vitro studies suggest hepatic fructose overload has direct effects on endoplasmic reticulum (ER) stress and inflammation that may lead to insulin resistance [10]. However, it has also been suggested that the increased hepatic lipid supply resulting from upregulated DNL and inhibited fat oxidation leads to increased levels of lipids such as diacylglycerol (DAG) that decrease hepatic insulin sensitivity by interfering with insulin receptor activation [41]. The increased hepatic lipid supply may also promote increased production and secretion of very low-density lipoprotein (VLDL) contributing to increased levels of circulating triglyceride (TG) and cholesterol [42]. Hepatic insulin resistance further promotes VLDL production and secretion by increasing apolipoprotein B (apoB) [43] and apoCIII [44] availability/expression. The ability of the insulin resistant liver to stimulate glycogen synthesis and suppress glycogenolysis is impaired [45], which then increases circulating glucose and compensatory insulin secretion. The compensatory hyperinsulinemia [46] and/or the chronic oversupply of substrate [45] continue to drive DNL.

Sustained increases of circulating TG may contribute to muscle lipid accumulation leading to impaired muscle insulin action and lowered whole body insulin sensitivity [47]. Fructose consumption may also impair glucose transport into muscle and adipose tissue [48,49], which could be a direct effect of fructose or one mediated by high levels of lactic acid decreasing expression of GLUT4 [50,51]. Uric acid may further contribute to the metabolic dysregulation by activation of fructokinase and induction of mitochondrial oxidative stress [52,53,54,55].

Our previously published paper, reporting dose-dependent increases in uric acid, TG, apoCIII, low density lipoprotein cholesterol (LDL-C), and apoB in young adults consuming 0, 10, 17.5, or 25% Ereq HFCS for two weeks [56], provides mechanistic support for HFCS as a mediator of the metabolic dysregulation described above. However, dose-dependent effects of HFCS on critical nodes in the progression of metabolic disease, specifically increased hepatic lipid content and decreased insulin sensitivity, have not been documented. Therefore, we hypothesized that two weeks of consumption of HFCS-SB at 0, 10, 17.5, or 25% Ereq would result in dose-dependent increases of hepatic lipid content and dose-dependent decreases in insulin sensitivity in the same young men and women [56]. In addition, to determine if our results supported the mechanisms outlined above, we conducted preliminary mediation analyses. We specifically sought to determine if the changes in hepatic lipid content potentially mediate the effects of HFCS dose on insulin sensitivity and on circulating TG concentrations. We also sought to determine if outcomes of other metabolic pathways directly affected by hepatic fructose overload, specifically increased uric acid and lactate concentrations, were potential mediators of hepatic lipid accumulation or decreased insulin sensitivity.

## 2. Materials and Methods

### 2.1. Participants

The current paper reports the results from 85 participants consuming three sweetened beverages per day containing either 0% (*n* = 23), 10% (*n* = 18), 17.5% (*n* = 16), or 25% (*n* = 28) of energy requirements (Ereq) from HFCS. The participants are a subgroup from an NIH-funded investigation that included 187 participants allocated to eight experimental groups. The results demonstrating the dose-response effects of HFCS-SB on circulating lipid/lipoprotein and uric acid concentrations from these 85 participants have been previously published [56]. Additionally, hepatic lipid content and insulin sensitivity results from the 28 participants who consumed 25% Ereq-HFCS and the 23 participants who consumed 0% Ereq-aspartame were recently reported [22]. Relevant results from this publication [22] are reported here in order to assess the dose-response effects of consuming 0, 10, 17.5, or 25% HFCS-SB on hepatic lipid content and insulin sensitivity.

This study was conducted in accordance with an experimental protocol that was approved by the University of California, Davis Institutional Review Board and is registered with Clinical Trials.gov (accessed on 7 November 2008): NCT0110392. Participants were recruited through a Craigslist.com listing and local flyer advertisements. Eligibility was assessed through telephone and in-person interviews followed by a complete blood count and serum biochemistry panel. All participants provided written informed consent. Inclusion criteria included males and females aged 18–40 years with a body mass index (BMI) range of 18–35 kg/m^2^ and a self-reported stable body weight of at least six months. Exclusion criteria included: diabetes (fasting glucose > 125 mg/dL), evidence of renal or hepatic disease according to aspartate aminotransferase (AST) and alanine aminotransferase (ALT) 1.5 normal limits ratio, fasting plasma TG > 400 mg/dL, hypertension (>140/90 mmHg), hemoglobin < 8.5 g/dL, and surgery for weight loss. Individuals were also excluded if they smoked, habitually ingested more than two alcoholic beverages/day, exercised more than 3.5 h/week at an intensity greater than walking, or used thyroid, lipid-lowering, glucose-lowering, anti-hypertensive, anti-depressant, or weight loss medications. By design, experimental groups were not randomized, but instead matched for sex, BMI, and circulating fasting TG, LDL-C, high-density lipoprotein cholesterol (HDL) and insulin concentrations measured in fasted serum collected during the in-person interviews. Five weeks before the start of study, enrolled participants were asked to limit their consumption of sugar-containing beverages to no more than one 8-oz serving of 100% fruit juice per day and to stop the use of any vitamin, mineral, dietary, or herbal supplements. Participant enrollment is detailed in a previous publication with the same experimental groups [56].

### 2.2. Dietary Protocol

**Figure 1:**
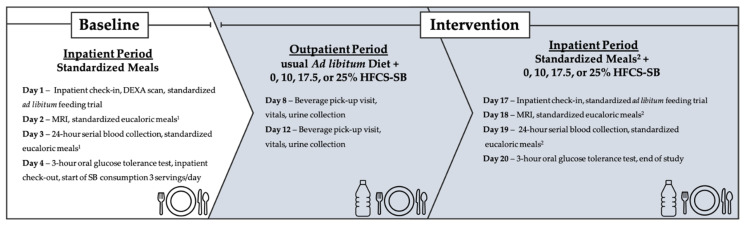
Study design and experimental testing and collection days. DEXA, dual energy X-ray absorptiometry; MRI, magnetic resonance imaging; SB, sweetened beverage; HFCS, high fructose corn syrup. ^1^ Fifty-five percent complex carbohydrate (<2%added sugar), 35% fat, 15% protein. ^2^ 55–30% complex carbohydrate (depending on assigned SB, <2%added sugar), 35% fat, 15% protein. % = % of energy requirement.

As previously described [22], this was a double-blinded, parallel-arm diet intervention study with three phases (Figure 1). The first phase included a 3.5-day inpatient baseline period at the University of California Davis Clinical and Translational Science Center’s Clinical Research Center (CCRC), where participants consumed a standardized low sugar diet and participated in experimental procedures. The second phase consisted of a 12-day outpatient intervention period where participants consumed their designated SBs providing 0% (aspartame-sweetened) or 10%, 17.5%, or 25% Ereq-HFCS along with their usual *ad libitum* diets. Phase three included a 3.5-day inpatient intervention period at the CCRC, where participants consumed a standardized diet that included their designated SBs and repeated all experimental procedures. Participants were instructed to maintain their usual physical activity level throughout the study. Fasted body weight was measured during baseline (Day 1) and intervention (Day 17) during inpatient testing periods at the CCRC with standardized attire.

#### 2.2.1. Inpatient Meals

Identical low sugar *ad libitum* meals were provided to all participants on Days 1 (baseline) and 17 (intervention) [57]. During Days 2 and 3 of the baseline inpatient period, energy-balanced meals consisting of 55% Ereq as predominantly low fiber complex carbohydrate (i.e., white bread, rice, and pasta), 30% fat, and 15% protein were provided. The meals provided on Days 18 and 19 of the intervention inpatient period were matched as closely as possible to baseline meals, including for fiber content, except for the isocaloric replacement of refined complex carbohydrate with the assigned HFCS-SB. The timing of inpatient meals and the energy distribution were: Breakfast 09:00-h (25% Ereq); Lunch 13:00-h (35% Ereq); Dinner 18:00-h (40% Ereq). Daily Ereq was calculated using the Mifflin equation with a 1.3 adjustment for physical activity on the days of the 24-h serial blood collections, and a 1.5 adjustment for the other days [58]. Participant-specific meal formulations were generated and provided to the kitchen staff by the Study Supervisor, who did not interact with participants. Meals were served by the Study Coordinator and CCRC nurses who were blinded to group assignment.

#### 2.2.2. Study Beverages and Outpatient Meals

Beverages were prepared by a designated staff member, who did not interact with participants, at the UC Davis, Department of Nutrition Ragle Clinical Research Center. All HFCS-containing beverages were flavored with an unsweetened powdered drink mix (Kool-aid^®^, Kraft Inc., Chicago, IL, USA) and sweetened with HFCS-55 (Isosweet 5500, 55% fructose, 45% glucose, Skidmore Sales and Distributing, Cincinnati, OH, USA), and 0% HFCS-containing beverages were prepared using a fruit-flavored aspartame drink mix (Market Pantry^®^, Target Brands Inc, Minneapolis, MN, USA). The aspartame drink mix was also used to increase the sweetness of the 10% Ereq-HFCS beverages. Study participants were blinded to their beverage assignment and voluntary feedback indicated that they were unable to distinguish between beverages containing aspartame or HFCS. Beverage amounts (grams) were standardized among the four groups and based on individual Ereq. During the 12-day outpatient intervention period, participants were instructed to drink one serving of study beverage with each meal of their usual diet and to not consume other sweetened beverages, including 100% fruit juice and fruit drinks. To monitor compliance, the study beverages contained a biomarker (0.015 mg riboflavin/mL; mean intake 16 mg riboflavin/day) that was measured fluorometrically in urine samples collected at the CCRC and during beverage-pickup appointments. Participants were informed of the biomarker in the study beverages but not its identity. Fasting urinary riboflavin concentrations were assessed in samples collected on Days 8, 12, and 17–20. Concentrations did not differ among groups, suggesting comparable compliance among all groups. Concentrations during or following outpatient beverage consumption (Days 8, 12, 17) were not different from those during monitored beverage consumption at the CCRC (Days 18–20), suggesting comparable compliance during the intervention outpatient and inpatient periods [56,59].

### 2.3. Hepatic Lipid Imaging

As previously described [22], on Day 2 (baseline) and Day 17 (intervention) of inpatient periods, magnetic resonance imaging (MRI) for hepatic lipid content was performed. Participants were scanned using a confounder-corrected chemical shift-encoded hepatic fat quantification MRI technique on a 1.5-Tesla system (General Electric Healthcare, Milwaukee, WI, USA, 1.5 T HDtx system, with an 8-channel body coil). To estimate the liver proton density fat fraction (PDFF), a quantitative image biomarker of liver fat content [60], axial 2D, T_1_-independent, T_2_*-corrected, 6-echo gradient-recalled-echo images were acquired (repetition time [TR] = 125 ms, echo time [TE] = 2.3, 4.6, 6.9, 9.2, 11.5, 13.8 ms, 8 mm slice thickness, 256 × 192 matrix size).

#### Hepatic Lipid Content Quantification

Participants with missing or incomplete scans were not included in the analysis of hepatic lipid content, leaving a total of 75 participants out of 85. Of the 10 sets of missing scans (aspartame-SB = 3; 10% HFCS-SB = 2; 17.5% HFCS-SB = 0; 25%HFCS-SB = 5), one was due to participant discomfort with the procedure (aspartame-SB), one was due to corrupt file format (aspartame-SB), and nine due to scanner unavailability due to malfunction or scheduling issues. Thus, the sample sizes available for analysis of hepatic lipid content were: aspartame-SB = 20; 10% HFCS-SB = 16; 17.5% HFCS-SB = 16; 25% HFCS-SB = 23. As previously described [22], MRI-PDFF was quantified using OsiriX software (OsiriX MD versions 10 & 11; Pixmeo, Geneva, Switzerland) and the LIPO-Quant (Liver Imaging of Phase-interference related signal Oscillation and Quantification) algorithm [61]. This algorithm assumes exponential decay and incorporates a multi-peak spectral model [62,63]. The technologists performing the scans and the image analyst were blinded to participant group and data.

### 2.4. Insulin Sensitivity

Three-hour Oral Glucose Tolerance Tests (OGTT) were conducted following a 14-h overnight fast at the CCRC on Day 4 (baseline) and Day 20 (intervention). A fasting blood sample was collected at 8:00-h through an intravenous catheter. Blood samples were collected 30, 60, 90, 120, and 180 min following a 75 g oral glucose load. One participant in the 17.5% HFCS group left the study on Day 19 due to a family emergency, thus did not participate in the intervention OGTT. Plasma samples were analyzed for glucose concentrations using a YSI glucose analyzer (YSI Inc., Yellow Springs, OH, USA) and for insulin with radioimmunoassay (Millipore Inc., St. Charles, MO, HI-14K). Two-hour OGTT data were used to derive two separate indices of insulin sensitivity: 2-h Matsuda Insulin Sensitivity Index (ISI) [64] and Predicted M ISI [65]. The Predicted M ISI utilizes the oral glucose insulin sensitivity index [66] and includes an adjustment for BMI [65]. Due to missing OGTT samples, Predicted M could not be calculated for six subjects (aspartame-SB = 1; 10% HFCS-SB = 2; 17.5% HFCS-SB = 2, 25% HFCS-SB = 1). Three-hour OGTT data were used to calculate total area under the curve (AUC) for glucose and insulin by the trapezoidal method. Fasting plasma glucose and insulin concentrations from the OGTT were used to calculate homeostasis model assessment—insulin resistance (HOMA-IR) [67].

### 2.5. Twenty-Four Hour Serial Blood Collection

Twenty-four-hour serial blood samples were collected via an intravenous catheter on Day 3 (baseline) and Day 19 (intervention). Three fasted samples were collected at 8:00-h, followed by 29 postprandial samples collected at 30- to 60-min intervals until 8:00-h the following morning. An extra 6 mL of blood were collected in the morning (8:00-, 8:30-, 9:00-h) and at late evening time points (22:00-, 23:00-, 24:00-h). The additional plasma from the fasting morning samples and the late-evening samples were pooled separately and aliquoted into 24 identical samples. The timing of the postprandial pool collection was based on the late-evening postprandial TG peak concentrations observed in our previous study [26]. The plasma concentrations of TG, uric acid, glucose, insulin, and lactate were analyzed at all time points and calculated for total 24-h AUC using the trapezoidal method. Lactate, glucose, and insulin amplitudes (AMP) were calculated as the difference between the post-meal peak concentration minus the pre-meal nadir for each of the three meals, then averaged. ApoCIII concentrations were measured during fasting and late-evening postprandial periods. TG, uric acid, and apoCIII concentrations were measured with a Polychem Chemistry Analyzer (PolyMedCo Inc., Cortlandt Manor, NY, USA) with reagents from MedTest DX (HORIBA, Ltd., Kyoto, Japan). Lactate was measured concurrently with glucose with a YSI Analyzer (YSI Inc., Yellow Springs, OH, USA). All assays were conducted by technicians who were blinded to beverage group assignment. The intra- and interassay coefficient of variation for the assays were as follows: glucose 3.6%, 4.5%; insulin: 6.5%, 7.6%; TG: 2.2%, 7.2%; uric acid: 1.9%, 14.5%; apoCIII: 0.9%, 5.5%.

### 2.6. Statistical Analyses

Data were log transformed when the baseline or absolute change values were not normally distributed (Shapiro–Wilk). The absolute change (∆) at two weeks of intervention compared with baseline for each outcome was tested for a dose-response trend in a general linear model (SAS 9.3; SAS, Cary, NC, USA), adjusted for sex, metabolic syndrome risk factor (MSRF), and outcome at baseline, using HFCS dose (0, 10, 17.5, 25) as a continuous variable. Departures from linearity were tested with polynomial terms using the same model. MSRF scores were assessed by assigning a value of 1 to measured clinical outcomes outside of established cutoffs [68] (waist circumference ≥120 cm for men, ≥88 cm for women, and ≥90 cm for Asian men and ≥80 cm for Asian women [69]; blood pressure ≥130 mmHg for systolic or ≥85 mmHg diastolic; fasting plasma glucose ≥100 mg/dl; serum TG ≥150 mg/dl; serum HDL ≤40 for men and ≤50 for women) and 0 if not, then taking the sum. To test for differences between and within groups, the ∆ or %∆ of each outcome was also analyzed in a general linear model (SAS 9.3); adjusted for sex, MSRF, and outcome at baseline; with HFCS-group (1, 2, 3, 4) as a categorical variable. Significant changes from baseline concentrations were identified as least squares means (LS means) of ∆ different from zero, and significant differences between groups were identified by Tukey’s multiple comparisons test. Statistical significance was considered at *p* < 0.05. Mediation analyses that included adjustment for sex and MSRF were conducted between the absolute change of correlated outcomes that were both significantly affected by HFCS dose [70,71]. These analyses only included subjects without missing data points, leaving a total of 71 participants (aspartame = 20; 10% HFCS-SB = 15; 17.5% HFCS-SB = 14; 25% HFCS-SB = 22). The percent attenuation due to mediation was calculated as: (1—(R of HFCS dose effect in model that controls for mediator/R of HFCS dose effect in model that does not control for mediator)) × 100. A multivariate analysis of the Δhepatic lipid content that included HFCS dose, sex, MSRF, and all mediators/partial mediators was conducted. Data are reported as mean ± standard deviation (SD) in Table 1, while all other data are means ± standard error of the mean (SEM).

## 3. Results

**Figure 2:**
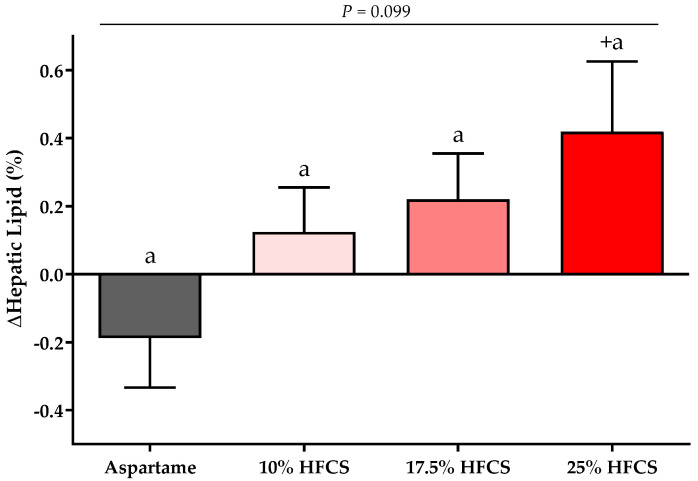
Changes in hepatic lipid content: Mean ± SEM of the absolute change (intervention − baseline) of hepatic lipid content in participants consuming 0 (*n* = 20), 10 (*n* = 16), 17.5 (*n* = 16), or 25% (*n* = 23) HFCS-sweetened beverages for two weeks. *p* = the effect of HFCS group, two-factor (HFCS group, sex) ANCOVA with adjustment for MSRF and outcome at baseline; ^+^ *p* < 0.05, LS mean different from zero.

**Figure 3:**
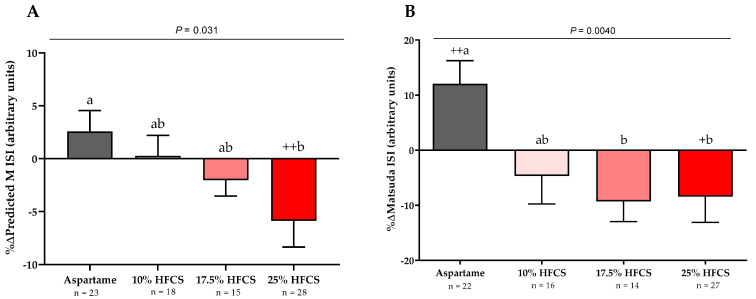
Changes in Predicted M ISI and Matsuda ISI: Mean ± SEM of the % changes in (**A**) Predicted M Insulin Sensitivity Index (ISI) in participants consuming 0 (*n* = 22), 10 (*n* = 16), 17.5 (*n* = 14), or 25% (*n* = 27) and (**B**) Matsuda ISI in participants consuming 0 (*n* = 23), 10 (*n* = 18), 17.5 (*n* = 15), or 25% (*n* = 28) HFCS-sweetened beverages for two weeks. *p* = effect of HFCS group, two-factor (HFCS group, sex) ANCOVA with adjustment for MSRF; ^+^ *p* < 0.5, ^++^ *p* < 0.01, LS mean different from zero; a different from b, Tukey’s.

**Figure 4:**
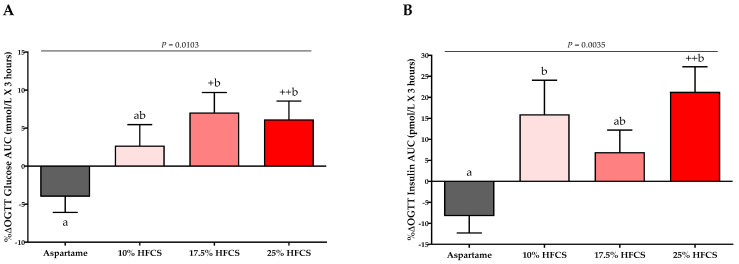
Percent changes in 3-h glucose and insulin AUC: Mean ± SEM of the % change in glucose (**A**) and insulin (**B**) AUC during OGTT in participants consuming 0 (*n* = 23), 10 (*n* = 18), 17.5 (*n* = 15), or 25% (*n* = 28) HFCS-sweetened beverages for the two-week intervention. *p* = effect of HFCS group, two-factor (HFCS group, sex) ANCOVA with adjustment for MSRF; ^+^*p* < 0.05, ^++^ *p* < 0.01, LS mean different from zero; a different from b, Tukey’s.

As previously reported [56] and shown in Table 1, there were no significant differences between the four experimental groups in baseline anthropomorphic or metabolic parameters. The absolute values for each outcome at baseline and intervention by HFCS dose group and the *p*-values for the effects of HFCS dose, sex, and MSRF are presented in Table 2. Table 2 includes body weight, which as previously reported, was significantly affected by HFCS dose, but was not significantly different between groups [56]. The absolute ∆ in hepatic lipid content is presented in Figure 2, and the %∆ in the insulin sensitivity indices (Matsuda, Predicted M ISI, 3-h OGTT AUC for glucose and insulin) are presented in Figure 3 and Figure 4, with notations depicting significant differences between and within groups.

### 3.1. Hepatic Lipid Content (MRI-PDFF)

There was a significant linear dose-response effect on hepatic lipid content (*p* = 0.016). MSRF significantly affected hepatic lipid content (*p* = 0.033) (Table 2), with participants with a higher MSRF score exhibiting greater increases. As shown in Figure 2, hepatic lipid content was significantly increased in participants consuming 25% HFCS-SB compared with baseline (*p* = 0.031).

### 3.2. Insulin Sensitivity

**Figure 5:**
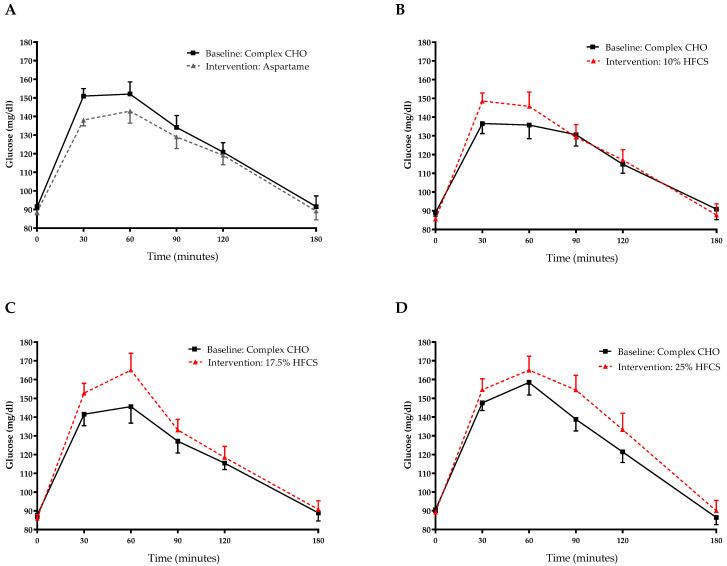
Plasma glucose excursions during 3-h OGTT: Glucose concentrations during OGTT at baseline and after consuming 0 (*n* = 23) (**A**), 10 (*n* = 18) (**B**), 17.5 (*n* = 15) (**C**), or 25% (*n* = 28) (**D**) HFCS-sweetened beverages for the two-week intervention. CHO, carbohydrate; HFCS, high-fructose corn syrup.

**Figure 6:**
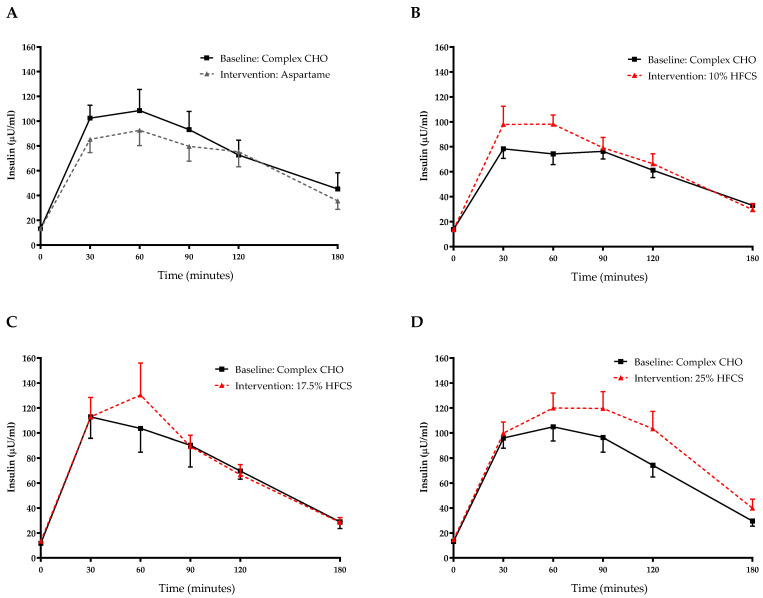
Plasma insulin excursions during 3-h OGTT: Insulin concentrations during OGTT at baseline and after consuming 0 (*n* = 23) (**A**), 10 (*n* = 18) (**B**), 17.5 (*n* = 15) (**C**), or 25% (*n* = 28) (**D**) HFCS-sweetened beverages for the two-week intervention.

The 3-h OGTT glucose and insulin responses at baseline and at the end of intervention are presented in Figure 5 and Figure 6. There were significant linear dose-response effects on the absolute changes in Matsuda ISI, Predicted M ISI, and on OGTT glucose and insulin excursions assessed as 3-h total AUC (Table 2). When analyzed as %Δ, the Predicted M ISI was significantly decreased in participants consuming 25% HFCS-SB compared with baseline (−5.9 ± 2.5%, *p* = 0.0036) and compared with participants consuming aspartame-SB (*p* = 0.022) (Figure 3A). Compared with baseline, the Matsuda ISI was significantly decreased in participants consuming 25% HFCS-SB (−8.4 ± 4.7%, *p* = 0.044) and significantly increased in participants consuming aspartame-SB (12.0 ± 4.2%, *p* = 0.0082; *p* = 0.0058, aspartame-SB vs. 25% HFCS-SB; *p* = 0.018, aspartame-SB vs. 17% HFCS-SB) (Figure 3B). OGTT glucose 3-h AUC (Figure 4A and Figure 5) increased significantly in participants consuming 17.5% (7.1 ± 2.6%, *p* = 0.023) and 25% HFCS-SB (6.1 ± 2.4%, *p* = 0.0062) compared with baseline and compared with participants consuming aspartame-SB (*p* = 0.031; *p* = 0.014, respectively). The OGTT insulin 3-h AUC (Figure 4B and Figure 6) was significantly increased in participants consuming 25% HFCS-SB compared with baseline (21.3 ± 5.9%, *p* = 0.0014), and compared with participants consuming aspartame-SB (*p* = 0.022). OGTT insulin 3-h AUC was also increased in participants consuming 10% HFCS-SB compared with those consuming aspartame-SB (*p* = 0.039). Fasting glucose and insulin concentrations (data not shown) and HOMA-IR (Table 2) were not significantly affected by HFCS dose or group.

### 3.3. Post-Meal Lactate, Glucose and Insulin Concentrations

**Figure 7:**
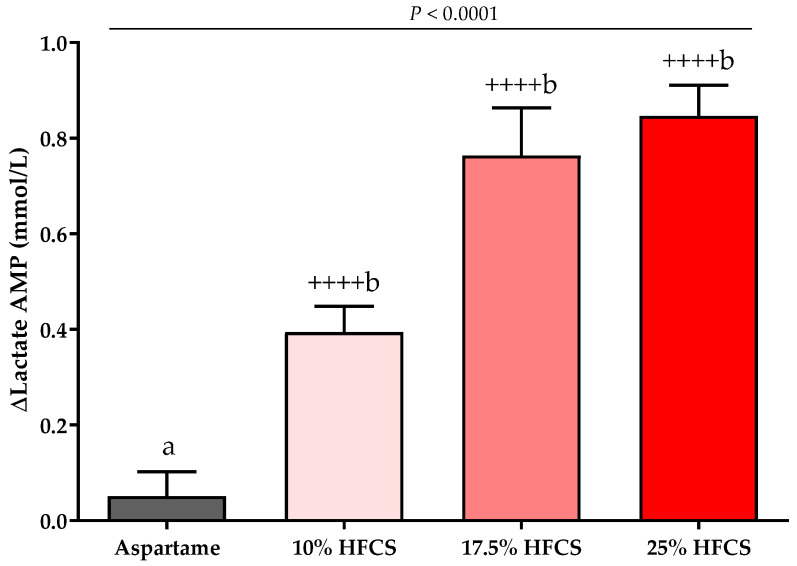
Changes in lactate amplitudes (AMP): Mean ± SEM of the absolute change (intervention − baseline) in lactate amplitudes in participants consuming 0 (*n* = 23), 10 (*n* = 18), 17.5 (*n* = 15), or 25% (*n* = 28) HFCS-sweetened beverages for the two-week intervention. *p* = the effect of SB group, two-factor (HFCS group, sex) ANCOVA with adjustment for MSRF and outcome at baseline; ^++++^ *p* < 0.0001, LS mean different from zero; a different from b, Tukey’s.

**Figure 8:**
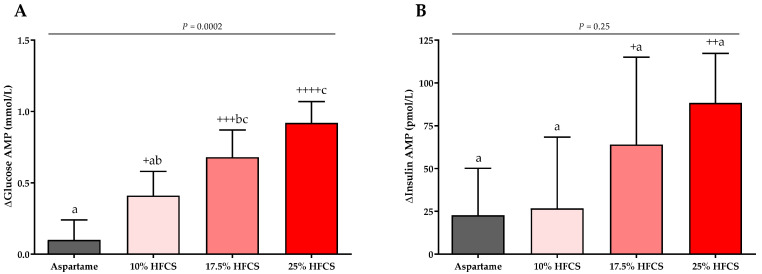
Changes in glucose and insulin amplitudes (AMP): Mean ± SEM of the absolute changes (intervention − baseline) in glucose (**A**) and insulin AMPs (**B**) in participants consuming 0 (*n* = 23), 10 (*n* = 18), 17.5 (*n* = 15), or 25% (*n* = 28) HFCS-sweetened beverages for the two-week intervention. *p* = effect of HFCS group, two-factor (HFCS group, sex) ANCOVA with adjustment for MSRF and outcome at baseline; ^+^ *p* < 0.05, ^++^ *p* < 0.01, ^+++^ *p* < 0.001, ^++++^ *p* < 0.0001, LS mean different from zero; a different from b, Tukey’s.

The baseline and intervention 24-h lactate, glucose, and insulin profiles are shown in the Supplement (Figures S1–S3). The profiles were quantified by both mean post-meal AMPs (Table 2, Figure 7 and Figure 8) and 24-h AUC (Supplement Table S1). Post-meal lactate AMPs were significantly affected by HFCS-dose (*p* < 0.0001, Table 2) and significantly increased in participants consuming 10, 17.5, or 25% HFCS-SB compared with baseline (all *p* < 0.0001) and compared with the aspartame-SB group (*p* = 0.0060, *p* = 0.0035, *p* < 0.0001, respectively) (Figure 7). The 24-h lactate AUCs were also significantly increased in participants consuming 17.5 or 25% HFCS-SB compared with those consuming aspartame-SB (Table S1). Post-meal glucose AMPs were significantly affected by HFCS-dose (*p* > 0.0001, Table 2), and significantly increased in participants consuming 10, 17.5, or 25% HFCS-SB compared with baseline (*p* = 0.014, *p* = 0.0002, *p* < 0.0001, respectively), and significantly increased in participants consuming 17.5 or 25% HFCS-SB compared with the aspartame-SB group (*p* = 0.044 and *p* < 0.0001, respectively) (Figure 8A). However, the post-meal glucose peaks following consumption of HFCS-SB were rapidly cleared such that the 24-h glucose AUCs remained unchanged in all groups (Supplement Table S1). As shown in Figure 8B, post-meal insulin AMPs were increased in participants consuming 17.5 or 25% HFCS-SB compared with baseline (*p* = 0.044 and *p* = 0.0029, respectively); however, there was no effect of HFCS-dose (*p* = 0.095, Table 2) or HFCS group (*p* = 0.25, Figure 8B).

### 3.4. Mediation Analysis

#### 3.4.1. Hepatic Lipid Content

The only index of ISI that was correlated with Δhepatic lipid content was the ΔOGTT insulin AUC (Table 3). As presented in Table 4, the mediation analysis suggested that ΔOGTT insulin AUC was a stronger partial mediator of Δhepatic lipid content than vice versa. The ΔOGTT insulin response attenuated the effect of HFCS dose on hepatic lipid content by 35% (from R = 0.26 to R = 0.17), while the effect of HFCS dose on ΔOGTT insulin AUC was attenuated by 15% and remained significant (*p* = 0.0068) when Δhepatic lipid content was controlled as the mediating variable.

When ΔapoCIII was controlled as the mediating variable, the effect of HFCS dose on Δhepatic lipid content was attenuated by 81%. As shown in Table 4, Δ24-h uric acid AUC, Δpost-meal glucose AMP, and Δbody weight attenuated the effects of HFCS dose on hepatic lipid content by 67, 40, and 33%, respectively. The effects of the partial mediators (OGTT insulin AUC, 24-h uric acid AUC, post-meal glucose AMP, and Δbody weight) on hepatic lipid content were independent of each other and of apoCIII. A model that included HFCS dose, MSRF, gender, and all the mediators accounted for 44% of the variation, with apoCIII explaining 16.6%, MSRF explaining 6.6%, and OGTT insulin AUC, Δbody weight, post-meal glucose AMP, and 24-h uric acid AUC and HFCS dose contributing 3.7, 2.7, 2.6, 1.2, and 0%, respectively.

There was no evidence that hepatic lipid content mediated the effects of HFCS dose on the changes in circulating TG. Rather, when Δ24-h TG AUC and postprandial TG were controlled as mediators, the effect of HFCS dose on hepatic lipid content was attenuated by 53% and 64%, respectively. However, when Δpostprandial apoCIII was included as a mediating variable along with Δ24-h TG AUC or postprandial TG, apoCIII remained a significant mediator of the effects of HFCS dose on hepatic lipid content and 24-h TG AUC and postprandial TG had no significant effects.

#### 3.4.2. Indices of Insulin Sensitivity

ΔPostprandial apoCIII was a partial mediator of ΔPredicted M ISI, attenuating the effect of HFCS dose by 48% (Table 4); however, it was not correlated with the other indices of insulin sensitivity. In contrast, the Δpost-meal lactate AMP was correlated with the changes in the Matsuda ISI and insulin and glucose OGTT AUC, but not Predicted M ISI. The Δpost-meal lactate AMP attenuated the effects of HFCS dose on ΔMatsuda ISI by 71% and partially attenuated the effect of HFCS dose on Δinsulin and glucose OGTT by 38% and 36% AUC, respectively.

## 4. Discussion

Insulin resistance is considered a central [72] or pivotal pathogenic component [73] of T2D and metabolic syndrome. NAFLD, diagnosed as hepatic lipid content exceeding 5% [74], has been described as the liver’s manifestation of metabolic syndrome [75,76]. This study provides novel results supporting our hypothesis that consumption of HFCS-SB at 10, 17.5, or 25% of Ereq increases hepatic lipid content and decreases insulin sensitivity in a linear dose-dependent manner. Demonstration of a dose-response relationship, also termed biological gradient, is considered strong evidence for a causal relationship between the exposure and the outcome [77,78]. The current results complement our previous reported results from the same participants showing dose-dependent increases in uric acid, triglyceride, apoCIII, LDL-C, and apoB [56].

A linear dose-response relationship between sugar-SB consumption and risk of NAFLD has been documented in observational studies [21]. A meta-analysis of twelve studies (case-control, cross-sectional or cohort studies), which included a total of 35,705 participants, showed consumptions of low doses (<1 cup/week), middle doses (1–6 cups/week), and high doses (≥7 cups/week) of sugar-SB increased the relative risk (RR) of NAFLD by 14%, 26%, and 53%, respectively (*p* = 0.01, *p* < 0.00001, *p* = 0.03, respectively) [21]. Similarly, a meta-analysis of nineteen prospective studies representing over a million participants showed a linear dose-response relationship between sugar-SB consumption and risk of T2D. The RR of T2D was 1.19 (95% CI 1.13–1.25) for each 250 mL/day of sugar-SB consumed [20]. Another recent meta-analysis reported linear dose-response relationships between sugar-SB and CVD incidence (RR 1.08, 95% CI 1.02 to 1.14 for each 250 mL/day) and risk of coronary heart disease (RR 1.15, 95% CI 1.09 to 1.22 for each 250 mL/day) [79]. Thus, the evidence from the observational studies [20,21,79] complements our current and previous results [56] and results from previous dietary studies [22,23,24,25,26,27,28,29,30,31,32,80], and together they provide a strong scientific foundation [81] to support the conclusion that excess sugar-SB consumption increases the risk of T2D, NAFLD, CVD, and metabolic syndrome.

This conclusion is further strengthened by the results from the first randomized controlled phase 2 clinical trial testing the administration of a fructokinase inhibitor in participants with NAFLD [82]. Relative to baseline, the fructokinase inhibitor (PF-06835919) led to a 26.5% reduction in liver fat, an 11.5% reduction in fasting uric acid, and a trend for improvements of other cardiometabolic parameters, including insulin resistance and inflammation [82]. This study documents the mechanistic role of fructokinase in mediating the unregulated hepatic fructose uptake and overload that leads to metabolic dysregulation. It is also worth noting that the favorable effects of the fructokinase inhibitor were accompanied by a small (<1 kg), but significant, increase of body weight [82]. This is in contrast to dietary intervention trials in which increases or decreases in fructose/sugar intake often induce parallel changes in risk factors and body weight, thus making it difficult to differentiate direct metabolic effects of sugar from those that are mediated by changes in body weight. However, in this study, preventing hepatic fructose overload via inhibition of fructokinase led to decreased liver lipid content and favorable metabolic effects that are clearly not confounded by favorable effects on body weight. This evidence [82] refutes the contention that dietary sugars are purely a highly palatable source of energy that have no unique or detrimental impact relative to any other source of calories [83].

We conducted simple mediation analyses to statistically test whether our data are indicative that the effects of HFCS dose on insulin sensitivity and circulating TG are mediated by the changes in hepatic lipid content. The results of these analyses did not support such a relationship with either outcome. Instead, with respect to insulin sensitivity, the increase in OGTT insulin AUC partially mediated the effects of HFCS dose on hepatic lipid content. This direction of partial mediation is plausible in that the hyperinsulinemia, which compensates for insulin resistance, activates hepatic lipid synthesis by increasing the activity of lipogenic enzymes [46,84]. Additionally, our data do not support previous work suggesting that hepatic lipid content regulates VLDL and TG production and secretion [42]. While the changes in hepatic lipid content were positively associated with the changes in circulating TG (24-h AUC and postprandial), there was no evidence that hepatic lipid content mediated the effects of HFCS dose on these outcomes.

The mediation analyses suggested that, in addition to OGTT insulin AUC, other potential mediators or partial mediators are implicated in the effects of HFCS dose on hepatic lipid content. ApoCIII was the strongest mediator of the change of hepatic lipid, attenuating the effect of HFCS dose by 81%. This relationship could be mediated directly, as it has been shown that an apoCIII gain-of-function variant enhances DNL and hepatic triglyceride-rich lipoprotein production [85], and overexpression of apoCIII increases hepatic lipid content in mice consuming low fat diets [86]. However, it is also possible that this relationship reflects the known effects of fructose (and glucose to a lesser extent) to upregulate both SREBP-1c and ChREBP [37,38]. These transcription factors increase both DNL and apoCIII production [37,38,39], thus, it may be enhanced lipid synthesis via DNL that mediates the effects of HFCS on hepatic lipid content. However, since DNL was not measured in the majority of the participants who participated in this study, we were unable to assess whether DNL mediated the effects of HFCS dose on hepatic lipid. The effect of HFCS dose on hepatic lipid content were also partially mediated by the changes in 24-h uric acid concentrations, post-meal glucose AMPs, and body weight. Experimental evidence suggests that uric acid can contribute to hepatic lipid deposition through induction of mitochondrial oxidative stress and increased expression of fructokinase [53,54,55], whereas evidence to suggest increased post-meal glucose AMPs promote accumulation of liver fat is lacking.

While it has been suggested that sustained increases in circulating TG may increase muscle lipid accumulation leading to impaired muscle insulin action and lowered whole body insulin sensitivity [47], we observed no associations between the indices of insulin sensitivity and circulating TG. Instead, the mediation analysis showed that post-meal lactate AMP attenuated the effects of HFCS on Matsuda ISI by 71%. Studies in rats in which lactate infusion inhibited glucose transport in skeletal muscle [51] and suppressed glycolysis and impaired insulin signaling [87] suggest potential mechanisms. Postprandial apoCIII attenuated the effect of HFCS dose on Predict ISI by 48%, possibly by inducing inflammation and inducing ER stress in muscle [88,89].

This is the first study to demonstrate that young men and women consuming HFCS-SB at 10, 17.5, or 25% of Ereq for two weeks exhibited dose-dependent increases in hepatic lipid content and dose-dependent decreases in insulin sensitivity. The only other published study that has examined the dose-dependent effects of sugar-SB consumption on hepatic lipid content and insulin sensitivity was funded by the Corn Refiners Association and generated null findings for both outcomes [90,91]. Reasons for the discrepant results have been previously discussed, both specifically [22,56,92] and generally [93].

The 2015–2020 Dietary Guidelines for America (DGA) recommends less than 10% of daily calories be consumed as added sugar [94]. The USDA 2019 loss-adjusted per capita caloric sweetener consumption is 344 kcal/day [13]. This suggests that the great majority of Americans are exceeding the Dietary Guideline’s recommendation for added sugars [94]. However, 344 kcal/day may underestimate the actual *per capita* intake because it is calculated based on the assumption that 34% of the caloric sweetener to which consumers are exposed is lost to spoilage, cooking loss, or plate waste. There is no question that it is difficult to accurately estimate food losses after the point of purchase. However, when comparing the spoilage potential, shelf life, and consumer preference of high sugar foods to that of fresh fruit, fresh vegetable, meat, and dairy products, it would seem intuitive that consumer losses of caloric sweeteners would be relatively lower. However, to obtain the USDA per capita consumption estimates, these are the corrections that are applied to various food groups to account for foods not consumed after the point of purchase; caloric sweeteners: 34%; fresh fruits and vegetables: 27%; dairy products: 23%; beef, pork, and lamb: 24%; fats and oils: 21%; grain products: 20% [13].

While the average consumption of sugar-SBs in the U.S. has decreased over the past ten years, consumption amounts vary greatly throughout the population. Between 2011 and 2014, it was found that more than two thirds of youths and one half of adults consume at least one sugar-SB on a given day, and at least 10% of youths and adults consumed at least two sugar-SB on a given day [17,18]. With a disproportionate number of heavy consumers being racial minorities from disadvantaged socioeconomic backgrounds, our results not only support dietary recommendations, but guide public health policies directed at decreasing health disparities [95].

### Study Strengths and Limitations

The use of an advanced MRI technique, providing a non-invasive and standardized quantitative biomarker suitable as a substitute for liver biopsy, for the quantification of hepatic lipid content is considered a study strength [96]. The presence of a biomarker in the study beverages as an objective measure of compliance is another strength of the study. Participants were informed of the biomarker and this awareness possibly contributed to the high degree of compliance evidenced by the consistent dose-response trends in this report and our previous publication [56]. In addition, conducting the baseline and intervention experimental procedures while subjects resided at the CCRC for 3.5 days and consumed standardized diets minimized the variability in the study results that can occur in outpatient settings due to non-compliance and differences in diet and physical activity.

A limitation of the study is that it was not randomized, which could have potentially introduced bias in the allocation of participants to the experimental groups. During the 12-day outpatient period, participants consumed the study beverages with *ad libitum* diets, therefore, the total amount of sugar consumed during this period cannot be accurately quantified. While the duration of the two-week intervention is relatively short, it demonstrates just how quickly excess sugar consumption can increase risk factors for cardiometabolic disease. A limitation of the mediation analyses is the smaller group sample sizes due to exclusion of participants with missing outcomes.

## 5. Conclusions

This study demonstrates that two weeks of consumption of HFCS-sweetened beverages providing 10, 17.5, or 25% Ereq results in dose-dependent increases in hepatic lipid content and dose-dependent decreases in insulin sensitivity in young men and women. These results contribute to the strong body of epidemiological, dietary intervention and mechanistic evidence that increased consumption of sugar-SB heightens risk for NAFLD and T2D.

## Figures and Tables

**Table 1:** Participants’ characteristics at baseline.

Parameters	Aspartame	10% HFCS	17.5% HFCS	25% HFCS
**Age (years)**	25.4 ± 6.2 ^1^	27.7 ± 6.0	24.1 ± 5.0	26.8 ± 6.6
**Sex (M/F)**	11/12	9/9	7/9	15/13
**Weight (kg)**	71.8 ± 10.6	70.9 ± 10.3	69.9 ± 14.3	72.9 ± 14.5
**BMI (kg/m^2^)**	24.8 ± 3.3	24.9 ± 3.8	24.2 ± 3.3	24.9 ± 4.0
**Waist circumference (cm)**	75.2 ± 6.4	76.2 ± 9.3	73.3 ± 7.7	77.0 ± 10.1
**Body fat (%)**	27.1 ± 9.8	26.8 ± 9.8	25.9 ± 9.9	26.0 ± 9.7
**Energy requirement** **(kcal/d)**	2354 ± 322	2323 ± 247	2326 ± 375	2390 ± 350
**Systolic blood pressure** **(mmHg)**	112.4 ± 11.5	114.9 ± 9.8	114.9 ± 8.4	117.1 ± 10.0
**Diastolic blood** **pressure (mmHg)**	69.2 ± 8.6	72.8 ± 8.2	71.3 ± 4.7	72.7 ± 7.2
**FST triglyceride (mmol/L)**	1.1 ± 0.6	1.4 ± 0.8	1.2 ± 0.6	1.2 ± 0.6
**FST total** **cholesterol (mmol/L)**	3.9 ± 0.7	4.2 ± 0.7	4.3 ± 0.9	4.1 ± 0.9
**FST** **HDL-cholesterol (mmol/L)**	1.02 ± 0.19	1.14 ± 0.29	1.20 ± 0.24	1.18 ± 0.35
**FST glucose (mmol/L)**	5.02 ± 0.37	4.96 ± 0.31	4.98 ± 0.34	5.03 ± 0.35
**FST insulin (pmol/L)**	88.2 ± 37.5	83.3 ± 23.6	79.2 ± 20.8	90.3 ± 37.5
**MSRF**	1.2 ± 1.0	0.9 ± 0.8	0.7 ± 1.0	1.1 ± 1.0
**FST AST (U/L)**	23.1 ± 8.4	25.3 ± 8.5	22.1 ± 5.2	22.1 ± 5.6
**FST ALT (U/L)**	23.4 ± 20.1	22.2 ± 5.9	20.6 ± 8.1	21.5 ± 8.9

^1^ Values are mean ± SD; HFCS, high fructose corn syrup; FST, fasting; MSRF, metabolic syndrome risk factor; AST, aspartate aminotransferase; ALT, alanine aminotransferase.

**Table 2:** Outcomes at baseline and intervention.

Outcome	Aspartame (*n* = 23)	10% HFCS (*n* = 18)	17.5% HFCS (*n* = 16)	25% HFCS (*n* = 28)	Effect of	*p*-Value
**Hepatic Lipid Content (MRI-PDFF, %)**					**Dose ^2^**	0.016
**Baseline**	1.6 ± 0.8 ^1^	1.2 ± 0.2	1.4 ± 0.3	2.3 ± 0.8	**Sex ^3^**	0.13
**Intervention**	1.4 ± 0.7	1.3 ± 0.2	1.6 ± 0.3	2.8 ± 0.9	**MSRF ^4^**	0.033
**Predicted M ISI (arbitrary units)**					**Dose**	0.0072
**Baseline**	1.52 ± 0.06	1.54 ± 0.06	1.60 ± 0.06	1.52 ± 0.05	**Sex**	0.44
**Intervention**	1.55 ± 0.05	1.54 ± 0.05	1.56 ± 0.06	1.45 ± 0.07	**MSRF**	0.33
**Matsuda ISI (arbitrary units)**					**Dose**	0.0087
**Baseline**	3.6 ± 0.3	3.7 ± 0.2	3.5 ± 0.2	3.3 ± 0.2	**Sex**	0.67
**Intervention**	3.9 ± 0.3	3.5 ± 0.3	3.2 ± 0.3	3.0 ± 0.3	**MSRF**	0.59
**OGTT Glucose AUC (mmol/L × 3 h)**					**Dose**	0.0004
**Baseline**	20.9 ± 0.7	19.2 ± 0.8	19.1 ± 1.0	21.1 ± 0.7	**Sex**	0.75
**Intervention**	19.9 ± 0.7	19.7 ± 0.9	20.3 ± 1.2	22.5 ± 0.9	**MSRF**	0.99
**OGTT Insulin AUC (pmol/L × 3 h)**					**Dose**	0.0004
**Baseline**	1392 ± 184	1033 ± 65	1292 ± 162	1335 ± 106	**Sex**	0.89
**Intervention**	1229 ± 151	1188 ± 97	1376 ± 165	1625 ± 154	**MSRF**	0.31
**HOMA-IR (arbitrary units)**					**Dose**	0.11
**Baseline**	3.0 ± 0.2	3.0 ± 0.3	2.5 ± 0.2	3.0 ± 0.2	**Sex**	0.97
**Intervention**	2.9 ± 0.2	2.9 ± 0.3	2.8 ± 0.2	3.3 ± 0.3	**MSRF**	0.0087
**Lactate AMP (mmol/L)**					**Dose**	<0.0001
**Baseline**	0.68 ± 0.06	0.62 ± 0.06	0.69 ± 0.06	0.73 ± 0.06	**Sex**	0.66
**Intervention**	0.73 ± 0.04	1.01 ± 0.07	1.50 ± 0.11	1.60 ± 0.09	**MSRF**	0.61
**Glucose AMP (mmol/L)**					**Dose**	<0.0001
**Baseline**	1.77 ± 0.19	1.88 ± 0.18	1.90 ± 0.22	2.04 ± 0.18	**Sex**	0.17
**Intervention**	1.87 ± 0.19	2.30 ± 0.16	2.63 ± 0.18	2.96 ± 0.21	**MSRF**	0.69
**Insulin AMP (pmol/L)**					**Dose**	0.095
**Baseline**	544.5. ± 84.0	424.3 ± 46.5	525.0 ± 62.5	506.9 ± 45.1	**Sex**	0.80
**Intervention**	567.4 ± 75.7	450.7 ± 35.4	588.9 ± 54.2	595.9 ± 48.6	**MSRF**	0.19
**Body Weight (kg)**					**Dose**	0.017
**Baseline**	71.8 ± 2.2	70.9 ± 2.4	69.9 ± 3.6	72.9 ± 2.7	**Sex**	0.079
**Intervention**	71.7 ± 2.2	70.9 ± 2.4	70.2 ± 3.7	73.7 ± 2.8	**MSRF**	0.15

^1^ Values are mean ± SEM; ^2^ effect of dose in the primary statistical trend testing model; ^3^ effect of sex in same model; ^4^ effect of MSRF in same model; HFCS, high fructose corn syrup; AUC, area under the curve; HOMA-IR, homeostasis model of insulin resistance; FST, fasting; AMP, amplitude; MSRF, metabolic syndrome risk factor.

**Table 3:** The relationship between the absolute change of outcomes ^1^ significantly affected by dose ^2^.

		HFCS Dose	Hepatic Lipid Content	Predicted M ISI	Matsuda ISI	Glucose OGTT AUC	Insulin OGTT AUC	Uric Acid 24 h AUC	Post-Meal Lactate AMP	Post-Meal Glucose AMP	apoCIII PP	TG 24 h AUC	TG PP	Body Weight
**HFCS dose**	R	1.000	0.256	−0.285	−0.238	0.372	0.371	0.608	0.632	0.406	0.385	0.319	0.521	0.303
	*p*-value		0.034	0.018	0.049	0.002	0.002	<0.0001	<0.0001	0.001	0.001	0.008	<0.0001	0.011
**Hepatic Lipid Content**	R	0.256	1.000	−0.236	−0.232	0.148	0.263	0.308	0.119	0.276	0.547	0.440	0.337	0.290
	*p*-value	0.034		0.051	0.056	0.226	0.029	0.010	0.330	0.022	<0.0001	0.000	0.005	0.016
**Predicted M ISI**	R	−0.285	−0.236	1.000	0.672	−0.638	−0.582	−0.191	−0.192	−0.057	−0.382	−0.192	−0.215	−0.152
	*p*-value	0.018	0.051		<0.0001	<0.0001	<0.0001	0.115	0.114	0.643	0.001	0.115	0.076	0.213
**Matsuda ISI**	R	−0.238	−0.232	0.672	1.000	−0.502	−0.690	−0.096	−0.293	−0.123	−0.233	−0.016	−0.127	0.163
	*p*-value	0.049	0.056	<0.0001		<0.0001	<0.0001	0.435	0.015	0.314	0.054	0.898	0.299	0.182
**Glucose OGTT AUC**	R	0.372	0.148	−0.638	−0.502	1.000	0.597	0.193	0.295	0.249	0.163	0.126	0.168	−0.013
	*p*-value	0.002	0.226	<0.0001	<0.0001		<0.0001	0.113	0.014	0.039	0.181	0.302	0.168	0.916
**Insulin OGTT AUC**	R	0.371	0.263	−0.582	−0.690	0.597	1.000	0.157	0.305	0.161	0.181	0.055	0.196	−0.055
	*p*-value	0.002	0.029	<0.0001	<0.0001	<0.0001		0.199	0.011	0.187	0.136	0.652	0.107	0.656
**Uric acid 24 h AUC**	R	0.608	0.308	−0.191	−0.096	0.193	0.157	1.000	0.304	0.314	0.296	0.166	0.259	0.347
	*p*-value	<0.0001	0.010	0.115	0.435	0.113	0.199		0.011	0.009	0.014	0.172	0.032	0.003
**Post-meal Lactate** **AMP**	R	0.632	0.119	−0.192	−0.293	0.295	0.305	0.304	1.000	0.464	0.230	0.124	0.266	0.020
	*p*-value	<0.0001	0.330	0.114	0.015	0.014	0.011	0.011		<0.0001	0.058	0.311	0.027	0.868
**Post-meal Glucose AMP**	R	0.406	0.276	−0.057	−0.123	0.249	0.161	0.314	0.464	1.000	0.191	0.261	0.301	0.059
	*p*-value	0.001	0.022	0.643	0.314	0.039	0.187	0.009	<0.0001		0.116	0.030	0.012	0.628
**apoCIII** **PP**	R	0.385	0.547	−0.382	−0.233	0.163	0.181	0.296	0.230	0.191	1.000	0.723	0.640	0.252
	*p*-value	0.001	<0.0001	0.001	0.054	0.181	0.136	0.014	0.058	0.116		<0.0001	<0.0001	0.037
**TG** **24 h AUC**	R	0.319	0.440	−0.192	−0.016	0.126	0.055	0.166	0.124	0.261	0.723	1.000	0.772	0.285
	*p*-value	0.008	0.000	0.115	0.898	0.302	0.652	0.172	0.311	0.030	<0.0001		<0.0001	0.018
**TG** **PP**	R	0.521	0.337	−0.215	−0.127	0.168	0.196	0.259	0.266	0.301	0.640	0.772	1.000	0.184
	*p*-value	<0.0001	0.005	0.076	0.299	0.168	0.107	0.032	0.027	0.012	<0.0001	<0.0001		0.130
**Body weight**	R	0.303	0.290	−0.152	0.163	−0.013	−0.055	0.347	0.020	0.059	0.252	0.285	0.184	1.000
	*p*-value	0.011	0.016	0.213	0.182	0.916	0.656	0.003	0.868	0.628	0.037	0.018	0.130	

^1^ N = 71, includes only participants for whom all outcomes are available. ^2^ All analyses adjusted for sex and metabolic syndrome risk factors (MSRF). HFCS, high fructose corn syrup; TG, triglyceride; ISI, insulin sensitivity index; OGTT, oral glucose tolerance test; AUC, area under the curve; AMP, amplitude; PP, postprandial.

**Table 4:** Potential or partial mediator(s) of the effects of HFCS dose ^1,2^.

Outcome (Absolute Change)	R for Effect of HFCS Dose w/o Mediator	Mediator (Absolute Change)	R for Effect of HFCS Dose w/Mediator	Attenuation (%)
**OGTT Insulin**	0.371	**Hepatic Lipid Content**	0.314	15
**Hepatic Lipid Content**	0.256	**OGTT Insulin**	0.166	35
**Postprandial apoCIII**	0.385	**Hepatic Lipid Content**	0.25	35
**Hepatic Lipid Content**	0.259	**Postprandial apoCIII**	0.048	81
**Uric acid 24h AUC**	0.608	**Hepatic Lipid Content**	0.536	12
**Hepatic Lipid Content**	0.256	**Uric acid 24h AUC**	0.084	67
**Post-meal glucose AMP**	0.406	**Hepatic Lipid Content**	0.338	17
**Hepatic Lipid Content**	0.256	**Post-meal glucose AMP**	0.153	40
**Body weight**	0.303	**Hepatic Lipid Content**	0.23	24
**Hepatic Lipid Content**	0.256	**Body weight**	0.171	33
**Predicted M ISI**	−0.285	**Postprandial apoCIII**	−0.149	48
**Postprandial apoCIII**	0.385	**Predicted M ISI**	0.284	26
**Matsuda**	−0.238	**Post-meal lactate AMP**	−0.068	71
**Post-meal lactate AMP**	0.632	**Matsuda**	0.576	9
**OGTT Insulin**	0.371	**Post-meal lactate AMP**	0.23	38
**Post-meal lactate AMP**	0.632	**OGTT Insulin**	0.56	12
**OGTT Glucose**	0.372	**Post-meal lactate AMP**	0.238	36
**Post-meal lactate AMP**	0.632	**OGTT Insulin**	0.556	11

^1^*n* = 71, includes only participants for whom all outcomes are available. ^2^ All analyses adjusted for sex and metabolic syndrome risk factors (MSRF). HFCS, high fructose corn syrup; OGTT, oral glucose tolerance test; TG, triglyceride; apoCIII, apolipoprotein C; AMP, amplitude; AUC, area under the curve; ISI, insulin sensitivity index.

## Data Availability

Restrictions apply to the availability of some or all data generated or analyzed during this study to preserve subject confidentially. The corresponding authors will on request detail the restrictions and any conditions under which access to some data may be provided.

## Supplementary Materials

The following are available online at https://www.mdpi.com/article/10.3390/nu14081648/s1, Table S1: Twenty-four-hour glucose, insulin and lactate AUCs, Figure S1: Twenty-four hour plasma lactate concentrations, Figure S2: Twenty-four hour plasma glucose concentrations, Figure S3: Twenty-four hour plasma insulin concentration.
