# Supplementary material for: The Dose-Response Effects of Consuming High Fructose Corn Syrup-Sweetened Beverages on Hepatic Lipid Content and Insulin Sensitivity in Young Adults
Source: Nutrients. 2022 Apr 15;14(8):1648. doi: 10.3390/nu14081648 (PMC9030734; doi:10.3390/nu14081648)
Supplement: Supplementary file 1 [file nutrients-14-01648-s001.zip › nutrients-1655596-supplementary.pdf]

Table S1. Twenty-four hour glucose, insulin and lactate AUCs.

Outcome	Aspartame (n = 23)	10% HFCS (n = 18)	17.5% HFCS (n = 15)	25% HFCS (n = 28)	Effect of	*P*-value
**Lactate 24-h AUC (mmol/L x 24h)**						
**Baseline**	21.9 ± 0.8[1]	22.5 ± 1.0	22.1 ± 1.2	22.6 ± 0.9	**Dose**	0.018
**Intervention**	22.2 ± 0.8[a]	23.5 ± 0.8[ab]	24.9 ± 1.2[***ab]	24.2 ± 1.0[***b]	**Group**	0.036
**Glucose 24-h AUC (mmol/L x 24h)**						
**Baseline**	130.0 ± 1.7	128.6 ± 1.3	129.8 ± 1.4	131.4 ± 1.6	**Dose**	0.99
**Intervention**	129.1 ± 1.7	129.0 ± 1.8	128.4 ± 2.0	130.8 ± 1.7	**Group**	0.77
**Insulin 24-h AUC (pmol/L x 24h)**						
**Baseline**	5,689.8 ± 792.4	4,601.0 ± 293.9	5,014.8 ± 457.9	5,291.4 ± 476.3	**Dose**	0.605
**Intervention**	5,444.9 ± 672.3	4,464.6 ± 259.6	4,832.5 ± 445.5	5,228.3 ± 460.0	**Group**	0.93

[1] Values are mean ± SEM; AUC, area under the curve; HFCS, high fructose corn syrup; *** $p < 0.001$, LS mean different from zero; a different from b, Tukey's.

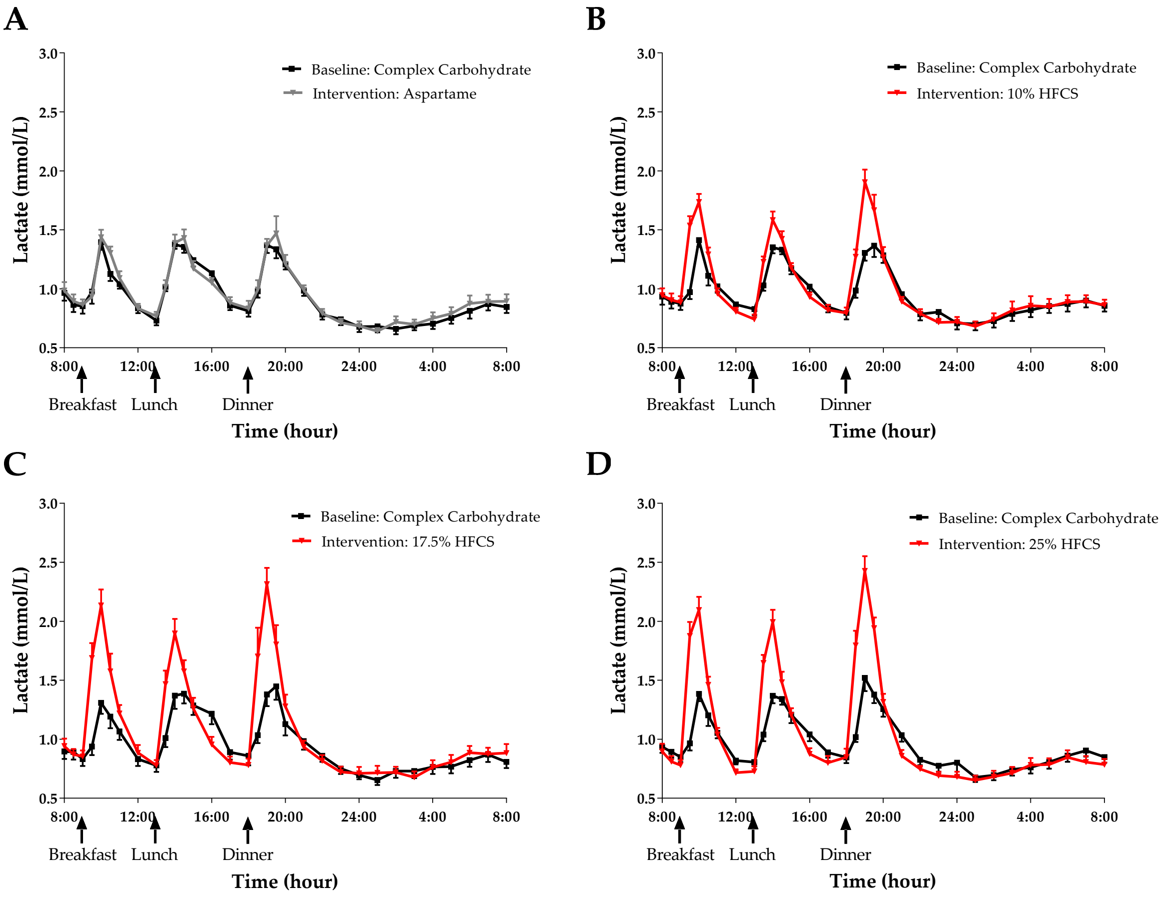

Figure S1. Twenty-four hour plasma lactate concentration at baseline and after consuming 0 (n = 23) (A), 10 (n = 18) (B), 17.5 (n = 15) (C), or 25% (n = 28) (D) HFCS-sweetened beverages for 2 weeks.

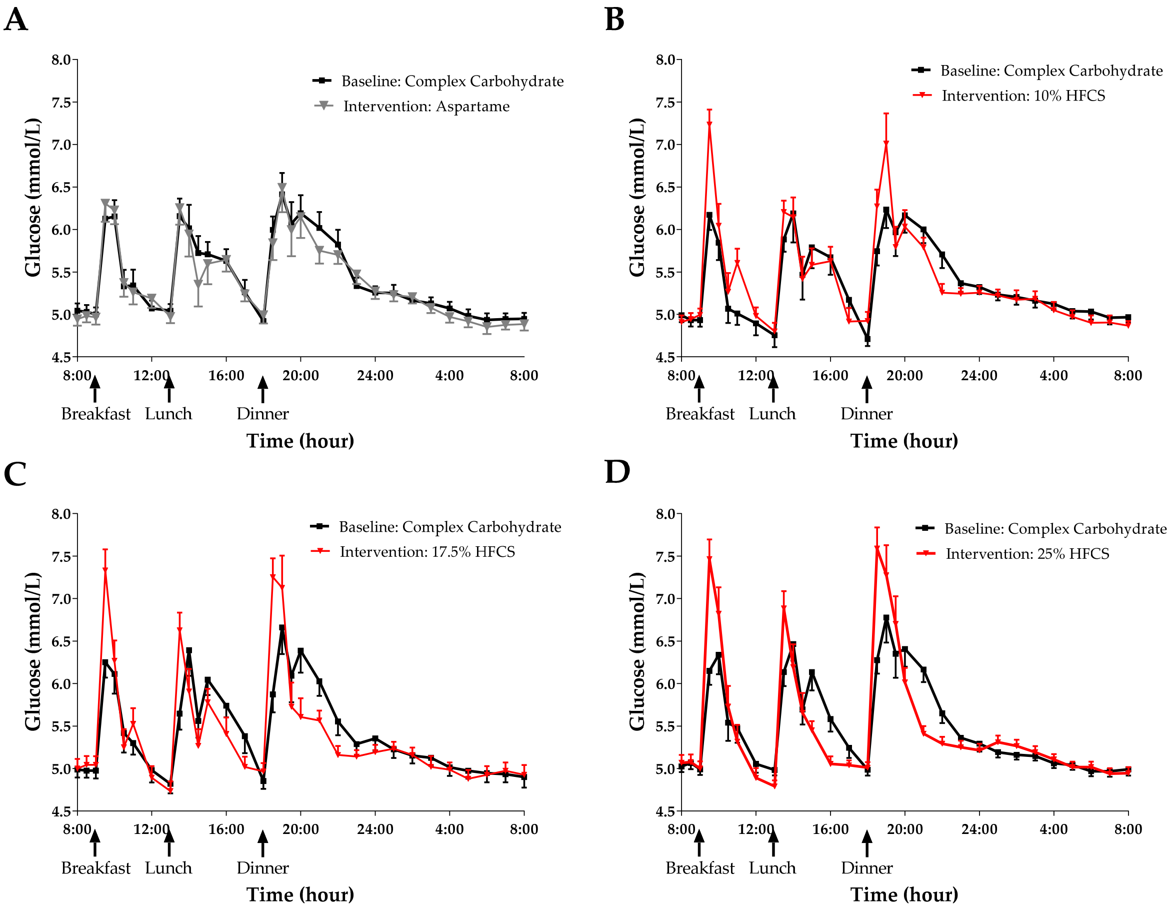

Figure S2. Twenty-four hour plasma glucose concentrations at baseline and after consuming 0 (n = 23) (A), 10 (n = 18) (B), 17.5 (n = 15) (C), or 25% (n = 28) (D) HFCS-sweetened beverages for 2 weeks.

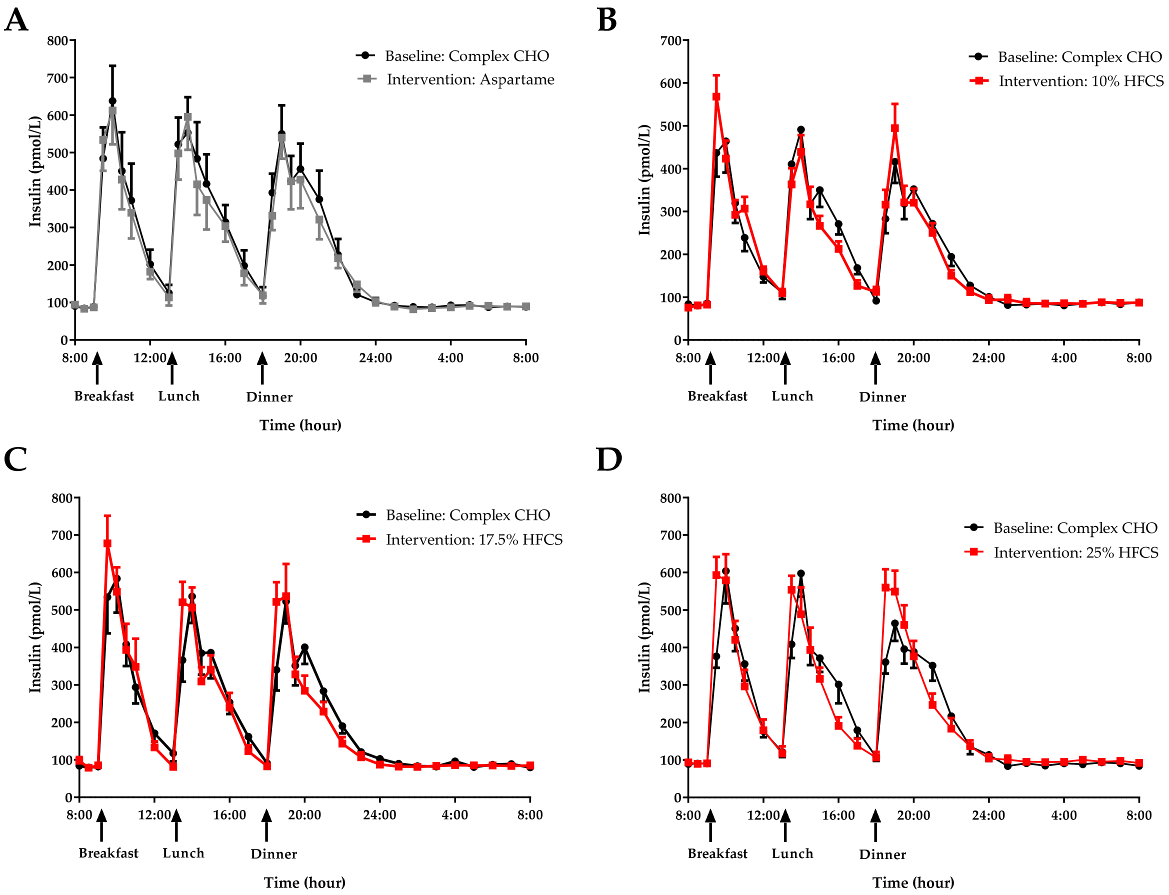

Figure S3. Twenty-four hour plasma insulin concentrations at baseline and after consuming 0 (n = 23) (A), 10 (n = 18) (B), 17.5 (n = 16) (C), or 25% (n = 28) (D) HFCS-sweetened beverages for 2 weeks.
